# A Comparative Analysis of Osteochondritis Dissecans and Avascular Necrosis: A Comprehensive Review

**DOI:** 10.3390/jcm13010287

**Published:** 2024-01-04

**Authors:** Wojciech Konarski, Tomasz Poboży, Klaudia Konarska, Andrzej Śliwczyński, Ireneusz Kotela, Jan Krakowiak

**Affiliations:** 1 Department of Orthopaedic Surgery, Ciechanów Hospital, 06-400 Ciechanów, Poland; tomasz.pobozy@onet.pl; 2 Medical Rehabilitation Center, Sobieskiego 47D, 05-120 Legionowo, Poland; klodiii87@gmail.com; 3 Social Medicine Institute, Department of Social and Preventive Medicine, Medical University of Lodz, 90-647 Lodz, Poland; andrzej.sliwczynski.ahe@gmail.com (A.Ś.); jan.krakowiak@umed.lodz.pl (J.K.); 4 Department of Orthopedic Surgery and Traumatology, Central Research Hospital of Ministry of Interior, Wołoska 137, 02-507 Warsaw, Poland; ikotela@op.pl

**Keywords:** osteochondritis dissecans, avascular necrosis, orthopedic, diagnosis, management

## Abstract

Musculoskeletal disorders, standing as the fifth leading cause of disability-adjusted life years globally, present significant challenges in orthopedics. Osteochondritis dissecans (OCD) and avascular necrosis (AVN) are distinct but closely related conditions within this spectrum, impacting patients’ quality of life with pain, limited mobility, and dysfunction. OCD, involving cartilage and bone detachment in joints, predominantly affects young athletes, but its exact etiology and optimal management remain subjects of ongoing research. Conversely, AVN, marked by bone tissue death due to compromised blood supply, is linked to systemic factors like corticosteroid use and traumatic injuries. Diagnosis for both conditions relies on radiography and magnetic resonance imaging. Conservative treatment for AVN includes the use of a cane or crutches, pharmacological therapy, or physical therapy. On the other hand, in OCD, the primary approach is activity/sports restriction. Surgical treatment options for AVN patients encompass core decompression, bone grafting, or, in the most advanced cases, total hip arthroplasty. OCD may be surgically treated through subchondral drilling or fixation of unstable lesions. Advanced cases of OCD involve cartilage salvage with resurfacing techniques. The presentation of differences between these conditions enhances our understanding, facilitating improved diagnosis and management strategies.

## 1. Introduction

Musculoskeletal disorders pose significant challenges in the field of orthopedics, affecting individuals of all ages and activity levels [1,2,3,4]. In 2017, musculoskeletal disorders ranked as the fifth leading cause of disability-adjusted life years (DALYs) worldwide and represented the primary contributor to global disability [5].

Among these disorders, osteochondritis dissecans (OCD) and avascular necrosis (AVN) stand out as two distinct but sometimes closely related conditions that can severely impact the quality of patients’ lives. Both conditions affect the integrity of bone and cartilage, often leading to pain, limited mobility, and long-term dysfunction [6,7,8,9,10]. De Smet [11] highlighted three key similarities between OCD and AVN. (1) The most frequent location for both types of lesions is the femoral condyles when the knee is affected. (2) In their advanced stages, both OCD and AVN can show similar radiographic features, such as the collapse of the subchondral bone plate, which can lead to the formation of intra-articular loose bodies. (3) Histologically, both conditions may present similarly with the presence of necrotic bone in the affected areas.

OCD, characterized by the detachment of cartilage and bone fragments within a joint, has long been a subject of clinical interest and debate. While it predominantly affects younger individuals, often athletes, and is believed to be linked to mechanical stress and repetitive trauma, its precise etiology and optimal management strategies remain topics of active research [8,9]. On the other hand, AVN, which most often occurs in the middle-aged and elderly, marked by the death of bone tissue due to compromised blood supply, is frequently associated with various factors, such as corticosteroid use, alcohol abuse, and traumatic injuries. Understanding the fundamental differences between these two conditions is vital for accurate diagnosis and timely intervention [6,7,11].

In this publication, we will explore the epidemiology and prevalence of OCD and AVN, considering the demographics and risk factors associated with each. We will also delve into the underlying pathophysiological mechanisms of the development and progression of these conditions. Additionally, we will highlight the similarities and differences in the clinical presentation of OCD and AVN and discuss the various diagnostic techniques and imaging modalities that aid in their accurate assessment.

This publication aims to provide a comprehensive comparative analysis of OCD and AVN, shedding light on their etiology, pathophysiology, clinical presentation, diagnostic modalities, and management strategies. By searching through the nuances and disparities between these two conditions, we seek to enhance our understanding of the unique challenges each presents and to improve the diagnosis and treatment of affected individuals.

## 2. Etiology

The specific pathomechanism of AVN is not well-known, and about 20% of cases are classified as idiopathic [12]. The initial pathological features of AVN include the necrosis of hematopoietic cells and adipocytes with subsequential interstitial bone marrow edema. Osteocyte necrosis manifests about 2 to 3 h after hypoxia, but the histological indications of osteocyte death do not emerge until around 24 to 72 h after oxygen deprivation [13,14,15,16].

Various factors contribute to AVN etiology, including underlying conditions or medications that increase the risk of vessel obstruction, alterations in osteocyte metabolism, and genetic factors [6,17]. 

Vascular impairment can result from both traumatic and non-traumatic factors such as intravascular occlusion and intraosseous extravascular compression [6,13,18]. 

Shah et al. categorized AVN risk factors into six groups: direct cellular toxicity, extraosseous arterial fracture, extraosseous venous, intraosseous extravascular compression, intraosseous intravascular occlusion, and multifactorial [13]. In traumatic cases, bone fractures or dislocations can obstruct the blood supply. In non-traumatic cases, the primary risk factors include treatment with corticosteroids, alcohol abuse, hyperlipidemia, autoimmune disease, Gaucher’s disease, sickle cell disease, and exposition to radiation [6,17]. According to recent meta-analysis findings, diabetes might elevate the risk of AVN in locations beyond the jaw, although the existing evidence is restricted. Larger, well-conducted, population-based studies are necessary to further explore this association [19]. 

There is also evidence that AVN of the femoral head may result from the surgical fixation of a femoral bone fracture [6,19,20,21]. The duration between surgery and injury appears pivotal in assessing the risk of bone ischemia, with a time interval exceeding 24 h linked to an increased risk [22].

Mueller-Weiss disease is a rare condition that involves spontaneous osteonecrosis of the tarsal navicular bone in adults. This disease is marked by the collapse of the navicular’s dorso-lateral part, leading to progressive fragmentation of the navicular and destruction of the talonavicular joint [23].

Initially, OCD was believed to result from inflammation occurring between the articular cartilage and subchondral bone. However, the current understanding of the disease suggests various potential causes, including spontaneous osteonecrosis, ischemia, genetic factors, and obesity [8,24]. It is now recognized that the most probable etiology of OCD is low-level repetitive trauma, particularly during sports activities [8,24]. The significant influence of sport-related microtrauma on elbow OCD has been extensively documented. Individuals who engage in sports that heavily involve the upper limbs, such as gymnastics, wrestling, baseball, and javelin, are at risk of developing elbow OCD [25,26,27]. OCD appears to start with a subchondral bone injury, gradually progressing through stages of bone resorption, eventual bone collapse, and the development of sequestration. Finally, this sequence may lead to the separation of the articular cartilage and the detachment of a subchondral bone fragment, culminating in the creation of a loose body [28].

There is a connection between childhood obesity and the presence of knee, ankle, and elbow OCD in children. According to a study by Kessler et al., extremely obese patients have an 86% higher risk of developing any form of OCD in comparison to those with normal weight. Furthermore, when considering specific types of OCD, extremely obese patients exhibited a 3.1 times greater risk of elbow OCD and a 3.0 times higher risk of ankle OCD [29]. The link between obesity and OCD in the elbow remains unclear. Contrary to weight-bearing joints, additional body weight is not typically anticipated to significantly elevate stress levels in the elbow joint [29].

Different anatomical variations, such as a posterior cruciate ligament footprint situated more distally and the presence of a discoid lateral meniscus, can also impact the mechanical conditions within the knee. Complete discoid menisci were linked to central OCD, whereas incomplete discoid menisci were associated with peripheral OCD [30,31,32]. Other researchers have shown a strong correlation between the varus axis and medial condyle OCD as well as between the valgus axis and lateral condyle OCD [33,34]. OCD of the talus occurs when a fragment of the osteochondral layer of the talar dome detaches in a patient who is still growing [35]. In the elbow, repeated stress on the lateral compartment may result in localized damage to the subchondral bone of the humeral capitellum, which has poor vascularization [36]. OCD of the hip and shoulder are less common. Shoulder OCD may involve the glenoid or the humeral head, while hip OCD involves the acetabulum or the femoral head [37]. 

Certain authors have proposed a familial inheritance role in the development of OCD, with instances of familial cases and a higher incidence among monozygotic twins. This implies the involvement of genetic factors related to cartilage turnover [8,38,39]. Yellin and colleagues [39] have identified specific genetic regions that may be important in understanding OCD pathophysiology. They found a cluster of genetic variations known as single-nucleotide polymorphisms (SNPs) on chromosome 13, as well as a top signal on chromosome 7, which may be critical for the coordinated expression of genes related to the condition. Furthermore, they highlighted an SNP on chromosome 12, specifically rs1464500, which is situated within the SOX5 gene. This gene is believed to influence the development of chondrocytes, which are essential for cartilage formation, potentially affecting cartilage development and contributing to OCD [40].

## 3. Epidemiology

AVN is most commonly diagnosed in individuals aged 30–65 [41]. There is limited data concerning AVN prevalence. According to a Swedish retrospective cohort study among people aged over 50 years, the incidence rate of AVN was 4.3 cases/100,000 person-years [42]. The most common locations for AVN are the femur, with 2.1 cases per 100,000 person-years, and the knee or lower leg, with 0.8 cases per 100,000 person-years. AVN is less common in other areas such as the ankle (0.3 cases per 100,000 person-years) and shoulder (0.1 cases per 100,000 person-years) [42].

The most significant risk factors associated with osteonecrosis included hip fracture with standardized incidence ratio (SIR) of 7.98; 95% CI, 7.69–8.27, solid organ transplantation (SIR, 7.14; 95% CI, 5.59–8.99), dialysis (SIR, 6.65; 95% CI, 5.62–7.81), and osteomyelitis (SIR, 6.43; 95% CI, 5.70–7.23) [42]. In the British case–control study, the incidence of AVN demonstrated a rising trend over the years, increasing from around 1.4 per 100,000 in 1989 to approximately 3 per 100,000 in 2003 [43].

The literature commonly reports the prevalence of knee OCD, with estimates ranging from 2.3 to 31.6 cases per 100,000 individuals [28,44]. In young individuals, the incidence of knee OCD increases from 6.8 per 100,000 among those aged 6 to 11, to 11.2 per 100,000 in the 12- to 16-year age group [44]. In adults, OCD occurs at a rate of 3.42 cases per 100,000 person-years, with the highest occurrence in ankles, and then knees. In contrast, juvenile OCD is more frequent, affecting 9.5 to 29 out of 100,000 knees, 2.2 out of 100,000 elbows, and 2 to 4.6 out of 100,000 ankles [44,45,46,47,48,49]. OCD predominantly affects young males, with a male-to-female ratio of 5:3. The disease is rarely seen before the age of 6, with the highest occurrence observed between the ages of 11 and 20 [28,50]. Some authors have reported an increased number of OCD cases in females, possibly due to the growing participation of women in sports [50]. Within a significant cohort of 1004 patients diagnosed with knee OCD, the majority of cases featured individuals who participated in multiple sports (68.1%). Among these cases, basketball was a predominant primary sport for males (27.3% of cases), while soccer held the same distinction for females (27.6% of cases) [51].

## 4. Clinical Presentation and Diagnosis

### 4.1. Clinical Picture

AVN most commonly affects the hip but also may occur in the humerus, knee, and talus. In rare cases, it may occur in the small bones of the wrist [7]. In the early stages of AVN, symptoms are often absent, and the standard physical examination tends to be normal, resulting in a delay in diagnosis [7]. Unfortunately, most patients do not present for evaluation until the AVN has reached later stages. Typical signs of AVN may manifest as growing pain, stiffness, and crepitus [6,52]. 

AVN of the hip is manifested by hip and groin pain and usually indicates late-stage progression. Associated symptoms can include referred pain in the buttock and thigh. The majority of patients have pain at rest. Others include stiffness and changes in gait [7,41]. 

AVN of the knee often manifests with acute pain during weight-bearing and at night. Typically, patients had a history of osteoporosis or osteopenia with no recent trauma. During the physical examination, pain is noted with palpation over the medial femoral condyle, accompanied by reduced range of motion [7,53].

AVN of the shoulder involving the proximal humerus is frequently linked to trauma and osteonecrosis in other parts of the body. The pain is described as pulsating, radiating to the elbow, with a noticeable decrease in the active range of motion [7].

Patients experiencing AVN of the talus often report gradually worsening ankle pain and a restricted range of motion [54].

AVN in the wrist typically occurs in the lunate and results in chronic, debilitating pain in the wrist [55].

The knee is the most commonly affected site by OCD followed by the ankle, elbow, shoulder, and hip [50]. Symptoms of OCD depend strictly on the localization of the disease and its stage. Stable lesions cause nonspecific symptoms such as swelling, pain, especially during activities or palpation, vague, crepitus, limited range of motion, and joint effusion. Unstable lesions or loose bodies may manifest by clicking or locking [35,45,56]. There are three main forms of OCD as follows [8]:

- Incidental finding in a symptom-free individual;
- Exercise-induced mechanical pain (most frequently observed);
- Persistent mechanical pain with joint swelling and/or locking.

### 4.2. Diagnosis

The correct diagnosis of AVN is the most accurate when a patient exhibits symptoms, imaging findings are positive, and other potential causes of pain and bone abnormalities are ruled out. In addition to clinical and physical examinations, diagnostic tools like radiographs and magnetic resonance imaging (MRI) scans are employed [41]. 

**Figure 1:**
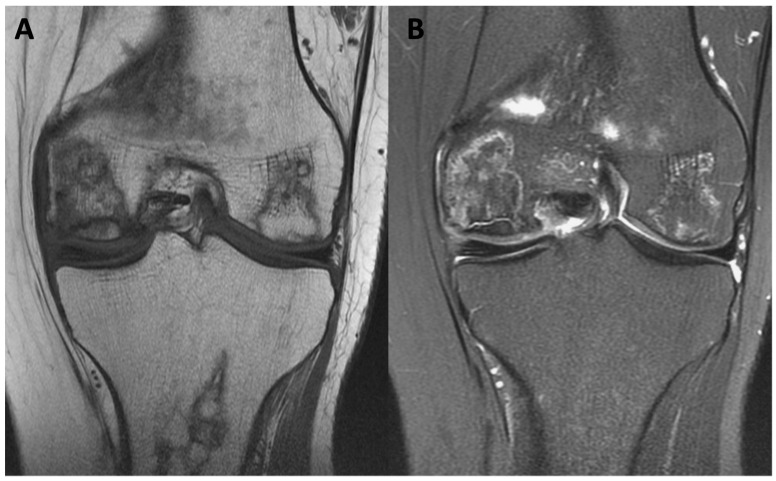
MRI image of AVN of the lateral and medial condyle of the femoral bone of the knee joint. (**A**) T1-weighted image; (**B**) short T1 inversion recovery (STIR) image.

**Figure 2:**
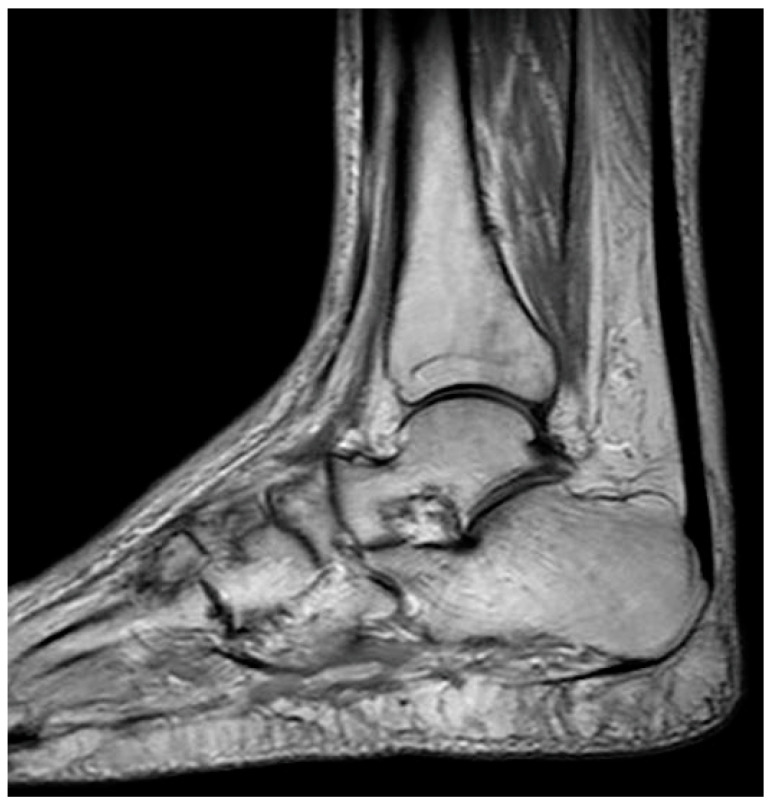
T1-weighted image of AVN of the talus bone.

**Figure 3:**
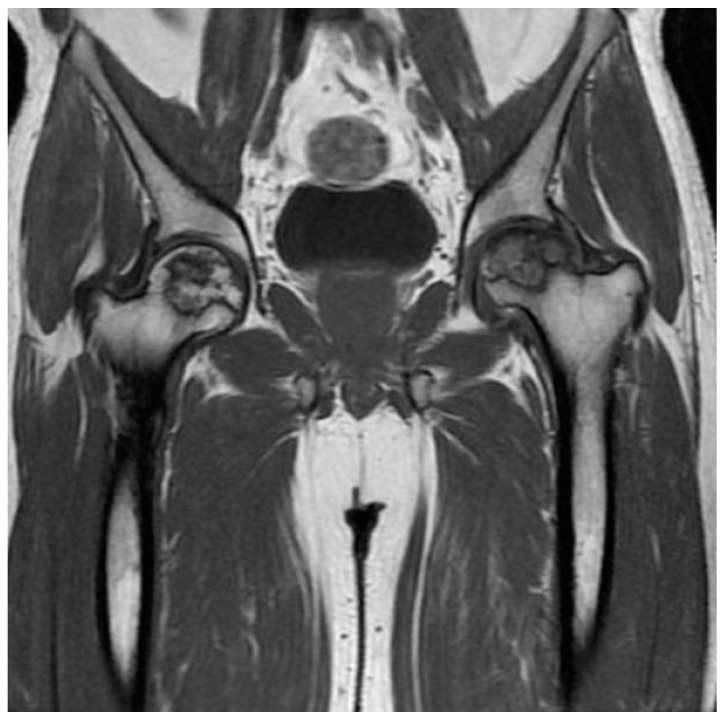
T1-weighted image of bilateral AVN of the femoral head.

The initial step in diagnosing AVN involves plain radiography followed by MRI [52]. Radiography is an affordable and widely accessible technique. The radiographic image may display subchondral radiolucency, referred to as the “crescent sign” suggesting subchondral collapse [57]. MRI stands out as the most precise method for diagnosing AVN. Bone marrow edema and joint effusion are important clinical prognostic factors [58]. In T1-weighted images, a single-density, low-signal intensity line is evident, whereas T2-weighted images display a high-intensity line with an early necrotic-viable bone interface [52,59]. Figure 1 displays MRI images of the knee with AVN, while Figure 2 illustrates the AVN of the ankle. Patients with a history of osteonecrosis should be monitored for bilateral AVN, which is reported in up to 70% of cases [52] (Figure 3). 

OCD can be identified via physical examination [45]. In cases of injuries to the medial condyle of the femur, the Wilson test elicits pain during knee extension from 90° to 30° with internal rotation of the tibia, and this discomfort diminishes with external rotation of the tibia [60]. Conversely, for capitellar joint injuries, the radiocapitellar compression test induces pain with active pronation–supination in full extension of the elbow joint [27]. The test results should be approached with caution as they have limited effectiveness [61].

Radiography is the initial diagnostic imaging method for OCD. Early lesions manifest as contour abnormalities, while more advanced lesions show a circumscribed ossified fragment separated from the bone by a radiolucent line [45]. Radiography may also be employed for monitoring the treatment of OCD. Since OCD may be bilateral in 14–30% of cases, radiography should be performed on both sides [62,63].

MRI is the preferred method for diagnosing OCD. It enables the accurate assessment of lesion size, bone edema, and the presence of an intra-articular loose body. In T1-weighted images, the progeny is typically hypointense, this sequence allows for a detailed analysis of the bone signal and provides measurements for lesion size. In T2-weighted images, the progeny is mostly heterogeneous; this sequence can assess the integrity of articular cartilage, reactive marrow edema in the parent bone, and fluid or cystic changes at the parent-progeny interface [45,64]. The existence of cysts at the interface between the progeny and parent bone indicates OCD chronicity [64].

It is crucial to note that in skeletally mature individuals (adults), MRI exhibits a high level of diagnostic sensitivity and specificity, reaching 100%. As a result, there is no controversy surrounding the use of MRI for confirming the diagnosis and assessing the necessity for surgery in adult patients with OCD [65]. In cases involving skeletally immature individuals (juveniles), MRI demonstrates a sensitivity of 100%, but its specificity is limited to 10–15%. Consequently, relying solely on MRI imaging for diagnosis and management in these patients is not advisable [65].

**Figure 4:**
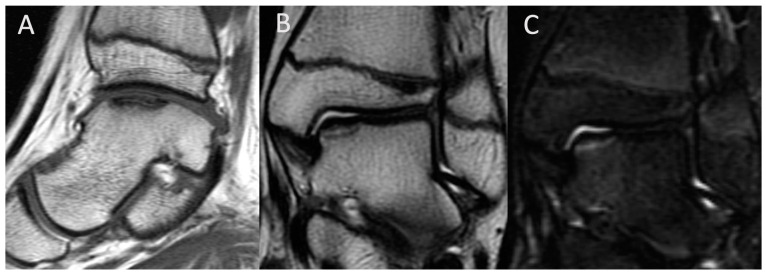
MRI image of talus OCD. (**A**) T1-weighted image (sagittal view); (**B**) T2-weighted image (coronal view); (**C**) short T1 inversion recovery (STIR) image (coronal view).

**Figure 5:**
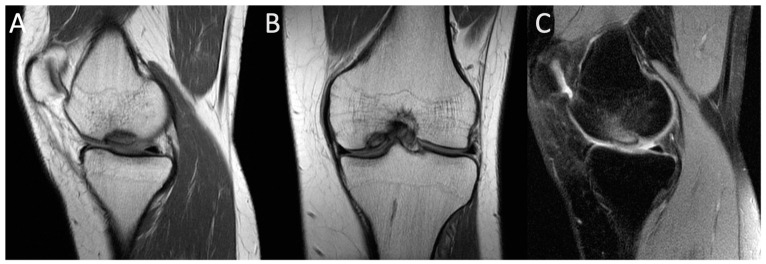
MRI image of medial condyle OCD. (**A**) T1-weighted image (sagittal view); (**B**) T1-weighted image (coronal view); (**C**) short T1 inversion recovery (STIR) image (coronal view).

Figure 4 and Figure 5 display MRI images of OCD in the ankle and medial condyle, respectively.

### 4.3. Differential Diagnosis

The differential diagnosis of AVN most commonly includes [6,59]:

- Osteoarthritis;
- Osteoarthritis secondary to acetabular dysplasia;
- Ankylosing spondylitis of the hip joint;
- Transient osteoporosis or bone marrow edema;
- Chondroblastoma of the femoral head;
- Incomplete fracture in subchondral bone;
- Pigmented villonodular synovitis;
- Synovial herniation;
- Femoroacetabular impingement syndrome;
- Bone infarction of the metaphysis.

The differential diagnosis of OCD in the pediatric population most commonly includes [66]:

- Patellofemoral syndrome;
- Patellar tendonitis;
- Osgood-Schlatter disease;
- Sinding-Larsen-Johannson syndrome;
- Fat pad impingement;
- Symptomatic discoid meniscus;
- Symptomatic synovial plica;

In the adult population, the following should be considered:;

- Patellofemoral pain;
- Knee osteoarthritis;
- Chondromalacia;
- Patellar tendonitis;
- Meniscal tear;
- Fat pad impingement;
- Symptomatic synovial plica.

### 4.4. Classifications

There are two primary classifications utilized in the diagnosis of AVN: the Ficat and Arlet classification and the Steinberg University of Pennsylvania classification. The first classification comprises four stages, determined by standard radiographs. Stage I denotes normal imaging. Stage II indicates a normal femoral head contour but with evidence of bone remodeling. Stage III signifies a “crescent sign” and/or evidence of subchondral collapse or flattening of the femoral head. Stage IV is end characterized by joint space narrowing flattening, femoral head collapse, and degenerative changes in the acetabulum [52,67]. 

Steiberg’s classification comprises six stages, with an evaluation of involvement in each stage. The classification enables differentiation between mild (<15% radiographic involvement of the femoral head), moderate (15–30% involvement of the femoral head), and severe (>30% involvement of the femoral head) stages [6,68].

There are two classifications of OCD severity based on radiography and MRI findings [8,69,70] (Table 1). 

## 5. Management

### 5.1. Nonsurgical Treatment

Nonsurgical treatment focuses on preventing bone collapse, delaying necrosis, and providing pain relief. It is primarily employed in patients with early-stage disease (Steinberg’s 0–1). The non-weight bearing, use of a cane, crutches, or a walker is one method to slow down disease progression [6,71,72,73]. The conservative treatment of humeral AVN involves lifestyle changes and avoiding intense shoulder movements like excessive abduction and flexion, while still maintaining flexibility through passive range of motion exercises [74].

Pharmacological treatment encompasses anticoagulants, statins, vasodilators, or bisphosphonates [73,75,76,77,78,79,80]. Enoxaparin is known to enhance arterial circulation and lessen hypoxia, potentially slowing down or reversing the effects of ischemic osteonecrosis [80]. In a similar vein, the bisphosphonate drug alendronate has demonstrated efficacy in reducing the occurrence of vertebral compression fractures, and it may also help prevent the deterioration of a femoral head affected by osteonecrosis [75]. Additionally, iloprost, a different medication, causes vasodilation, thereby improving microcirculation and boosting blood flow [79]. Regarding the treatment of AVN, statins work by inhibiting the formation of fat cells and encouraging the formation of bone cells. This is achieved through the downregulation of PPARγ2 and the upregulation of Cbfa1/Runx2 in bone marrow mesenchymal cells. The effect of statins is observed mainly in patients with corticosteroid-related AVN [77].

However, data on these drugs are limited, and specific guidelines for their use in AVN are lacking. Patients may also use pain relief drugs such as nonsteroidal anti-inflammatory drugs and acetaminophen [41]. 

Additional non-surgical therapies involve extracorporeal shockwave therapy or pulsed electromagnetic fields, which stimulate osteoblastic activity and alleviate pain [72,81,82,83]. Certain studies have reported positive outcomes with hyperbaric oxygen therapy in individuals with early-stage AVN. Hyperbaric oxygen enhances extracellular oxygen concentration, induces vasoconstriction, and reduces ischemia and edema [84,85].

Nonsurgical treatment for OCD is particularly intended for skeletally immature patients with stable lesions and minimal symptoms. The primary recommendation involves activity/sports restriction. Other procedures include immobilization through casting, bracing, splinting, weight-bearing restriction, and muscle-strengthening exercises. This form of treatment should be administered for a duration of 3–6 months [8,35,45]. Some authors have raised concerns about the consequences of prolonged immobilization, particularly regarding the development of stiffness [86,87]. Tepolt et al. suggested that short-term protected weight-bearing using crutches is likely advantageous. They also mentioned that the use of bracing could be based on the provider’s preference and a collaborative decision-making process involving the families [87].

Pharmacotherapy of OCD most often includes non-steroidal anti-inflammatory drugs [36].

### 5.2. Surgical Treatment

Surgical approaches for patients with AVN may differ based on whether it is in the early or late stages. In the early stages, core decompression is a common treatment method aimed at reducing intraosseous pressure and restoring normal blood flow. The method has evolved over time from single drilling to multiple drillings [88,89,90,91,92,93]. The number of drillings and trephine diameter should be chosen individually to minimize morbidity and the risk of bone weakening [94].

Bone grafts may be vascularized or nonvascularized and have various origins (allograft, autograft, or artificial). The procedure can be performed after core decompression or in combination using the core decompression tract. Vascularized grafting not only enhances subchondral architecture but also reinstates circulation in the affected area [95,96,97,98,99].

Total hip arthroplasty is considered for patients with significant femoral head collapse and a substantial decrease in quality of life. This method has shown good outcomes, and modern ceramic, low-wear, friction torques are a reasonable choice for young, active patients [100,101]. In patients with talus AVN, partial or total talar replacement has shown promising results [72]. Knee and humeral arthroplasty may also be successful treatments in advanced cases of AVN [102,103].

Surgical treatment for OCD is reserved for patients with unstable lesions after the failure of nonoperative treatment. In less advanced cases, subchondral drilling (transarticular or retroarticular) is commonly employed. This method stimulates the influx of mesenchymal cells and growth factors, leading to neovascularization and ossification of the cartilaginous lesion [35,36,37,104,105,106,107]. The results of OCD drilling are generally positive, showing high rates of healing and low complication rates in most cases [108,109].

Managing unstable OCD lesions involves articular surface restoration, fracture fixation, and vascular enhancement. Numerous techniques are available for fixing unstable OCD lesions, including screws, anchors, arrows, and pins, which can be implanted arthroscopically or during open surgery. Regardless, a critical step involves precise debridement for the removal of fibrotic tissue and drilling to create vascular channels, aiming to maximize revascularization and enhance the cure rate [8]. Fixation is recommended for detached osteochondral fragments, and a hybrid model, combining metallic or resorbable mechanical compression screws with osteochondral autograft transplantation, has been recently employed [8,36]. This procedure shows promising short-term outcomes with a low complication rate [37,110,111,112].

In large lesions with excessive fragmentation, fixation is not possible. In such cases, the affected fragment is removed, and cartilage salvage and resurfacing techniques are employed. These may include microfractures, autologous chondrocyte implantation, bone marrow stimulation, fresh osteochondral allografts, or autologous chondrocyte transplantation [8,45].

### 5.3. Biologic Treatment

Biological therapies for AVN primarily focus on treating the condition when it is present in the femur. The technique primarily involves the injection of mesenchymal stem cells or osteoinductive agents like bone morphogenic proteins. The goal of this approach is to initiate revascularization and regenerate bone [113,114]. It can be combined with core decompression, which has promising results [115]. The findings from two meta-analyses suggested that the combined use of core decompression and autologous bone marrow-derived mesenchymal stem cells is more effective in providing pain relief and improving clinical outcomes compared to core decompression alone in the treatment of osteonecrosis of the femoral head. Additionally, this combined approach can more effectively delay the collapse of the femoral head [116,117].

There is no evidence of the use of biological treatment in patients with OCD.

## 6. Conclusions

In conclusion, this comprehensive review has provided a thorough comparative analysis of OCD and AVN. By examining various aspects, including etiology, pathomechanisms, management, and other relevant factors, we have outlined key distinctions between these two orthopedic conditions. We included a detailed summary of these differences in Table 2. This analysis contributes to a better understanding of the distinct characteristics of OCD and AVN, aiding healthcare professionals in accurate diagnosis and effective management strategies for these conditions.

## Figures and Tables

**Table 1:** OCD severity classification based on radiography and MRI [8,69,70].

Stage/Type	Radiography [70]	[69]
I	Small lesion, compression of subchondral bone	Articular cartilage thickening and low signal alterations, but no fractures detected
II	Partially detached OCD fragment	Articular cartilage breached, with a low-signal rim behind the fragment indicating fibrous attachment.
III	Completely separated OCD fragment, still located in the underlying crater	Articular cartilage breached, with a high-signal rim behind the fragment indicating synovial fluid between the fragment and the underlying subchondral bone
IV	Full detachment or loose body	Loose body

Abbreviations: MRI—magnetic resonance imaging; OCD—osteochondritis dissecans.

**Table 2:** Summary of differences between AVN and OCD.

	AVN	OCD
Pathophysiology	Blockage and reduction in subchondral microcirculation leading to necrosis of hematopoietic cells and adipocytes with subsequential interstitial marrow edema and osteocyte death.	Subchondral bone injury progresses from resorption to collapse, leading to sequestration. This sequence may result in the separation of articular cartilage and the detachment of a subchondral bone fragment, forming a loose body.
Risk factors	Traumatic factors: Bone fractures or dislocations; Non-traumatic factors; Corticosteroids; Alcohol abuse; Hyperlipidemia; Surgical fixation of a femoral bone fracture; Autoimmune disease; Gaucher’s disease; Sickle cell disease; Radiation; Solid organ transplantation.	Low-level repetitive trauma, particularly during sports activities; Childhood obesity; Anatomical deviations; Genetic factors.
Incidence	0.1–4.3 cases/100,000 person-years—depending on the site	2.3–31.6 cases per 100,000 individuals–depending on the site
Most common sites	Hip; Humerus; Knee; Talus; Small bones of wrist.	Knee; Ankle; Elbow; Shoulder; Hip.
Age	Typically, 30–65 years old	Typically, 11–20 years old
Clinical manifestation	In the early stages of AVN, symptoms are often absent. Typical signs of AVN may manifest as growing pain, stiffness, and crepitus.	Stable lesions cause nonspecific symptoms such as pain, especially during activities or during palpation, vague, crepitus, limited range of motion, and joint effusion. Unstable lesions or loose bodies may manifest by clicking or locking.
Imaging	Radiography followed by MRI.	Radiography followed by MRI.
Radiographic features	Subchondral radiolucency, referred to as the “crescent sign” suggesting subchondral collapse.	Early lesions manifest as contour abnormalities. Advanced lesions show a circumscribed ossified fragment separated from the bone by a radiolucent line.
MRI features	T1-weighted images display a single-density, low-signal intensity line. T2-weighted images display a high-intensity line with early necrotic-viable bone interface.	In T1-weighted images, the progeny is typically hypointense. In T2-weighted images, the progeny is mostly heterogeneous; this sequence can assess the integrity of articular cartilage, reactive marrow edema in the parent bone, and fluid or cystic changes at the parent-progeny interface.
Rate of bilateral cases	Up to 70%	14–30%
Conservative treatment	The use of a cane, crutches, or a walker; Pharmacological treatment with anticoagulants, statins, vasodilators, or bisphosphonates; Extracorporeal shockwave therapy; Hyperbaric oxygen.	Activity/sports restriction; Immobilization through casting, bracing, splinting; Weight-bearing restriction.
Surgical treatment	Core decompression; Bone grafting (vascularized or not); Arthroplasty.	Subchondral drilling; Fixation of unstable lesions; Osteochondral autograft transplantation; Cartilage salvage with resurfacing techniques.

Abbreviations: AVN—avascular osteonecrosis; MRI—magnetic resonance imaging; OCD—osteochondritis dissecans.

## Data Availability

Not applicable.
